# The effect of ‘sleep high and train low’ on weight loss in overweight Chinese adolescents: study protocol for a randomized controlled trial

**DOI:** 10.1186/1745-6215-15-250

**Published:** 2014-06-25

**Authors:** Ru Wang, Dongmei Liu, Xueqiang Wang, Weihua Xiao, Nana Wu, Binghong Gao, Peijie Chen

**Affiliations:** 1 Key Laboratory of Exercise and Health Sciences of Ministry of Education at the Shanghai University of Sport, #650 Qingyuanhuan Road, Shanghai 200438, China

**Keywords:** Hypoxia, Living high-training low, Weight loss, Adolescents, Gastrointestinal hormone

## Abstract

**Background:**

Exercise and diet are the cornerstones for the treatment of obesity in obese children and adolescents. However, compensatory changes in appetite and energy expenditure elicited by exercise and dieting make it hard to maintain a reduced weight over the longterm. The anorexic effect of hypoxia can be potentially utilized to counteract this compensatory increase, thereby enhancing the success of weight loss. The purpose of the study is to assess the effectiveness of four week intermittent hypoxia exposure added to a traditional exercise and diet intervention on inducing short- and longterm weight loss in obese adolescents.

**Methods/Design:**

In this randomized parallel group controlled clinical trial, 40 obese adolescents (20 boys and 20 girls, 11 to 15-years-old), will be recruited from a summer weight loss camp at the Shanghai University of Sport, China. Participants will be stratified by gender and randomly assigned to either the control group or the hypoxia group. During the four-week intervention period, both groups will exercise and eat a balanced diet. Additionally, the control group will sleep in normal conditions, while the hypoxia group will sleep in a normobaric hypoxia chamber (sleep high and train low). The primary outcome will be body composition and the main secondary outcomes will be the circulating levels of appetite regulatory gastrointestinal hormones. All the outcome measures will be assessed at baseline, after the four-week intervention, and at two months follow-up.

**Discussion:**

Our study will be the first to evaluate the effectiveness of ‘sleep high and train low’ on short- and longterm weight loss among obese adolescents. A potential mechanism for the appetite regulatory effect of hypoxia will also be explored. The results of the study will provide an evidence-based recommendation for the use of hypoxia in a weight loss intervention among obese children and adolescents. Furthermore, the clarification of mechanisms leading to weight loss in ‘sleep high and train low’ might provide information for the development of new strategies in combating obesity.

**Trial registration:**

This trial was registered on 10 January 2014 at the Chinese Clinical Trial Registry with the registration number: ChiCTR-TRC-14004106.

## Background

Obesity is a global pandemic and its prevalence among children and adolescents has also increased worldwide, becoming a serious public health problem [1]. In China, nearly 215 million people are overweight or obese, and 12% of these are children (< 17-years-old), as estimated by the 2002 China National Nutrition and Health Survey [2]. Obesity in childhood has been associated with an increased risk of diabetes, hypertension, cardiovascular disease and various cancers in adulthood [3,4]. Therefore, preventing and treating obesity in children and adolescents is crucial.

The causes of childhood obesity are complex and multifaceted involving genetic factors, environmental, and behavioral factors. The current worldwide epidemic of obesity is believed to be attributable to the modern living environment which promotes a sedentary lifestyle and excessive consumption of calorie-dense food. Accordingly, a lifestyle modification including a healthy diet and increased physical activity has been recommended as the cornerstone of prevention and treatment of obesity. Physical activity increases energy expenditure and, in combination with a healthy diet, is effective in inducing weight loss. However, the reduced weight achieved through weight loss programs is hard to maintain over the longterm. The failure to achieve longterm weight loss is believed to be caused by compensatory changes in appetite and energy expenditure elicited by exercise and dieting. Currently, a complete understanding of the relationship between exercise, appetite regulation, and weight management is lacking.

Recent research has revealed that appetite-regulating hormones might play an important role in moderating the interrelationship between exercise and dieting, appetite, and weight regain. Among various potential appetite-regulating hormones, the gastrointestinal hormones, ghrelin, peptide YY (PYY), cholecystokinin (CCK) and glucagon-like peptide-1 (GLP-1) are well studied. Ghrelin is the only hormone that has been shown to be orexigenic, while PYY, CCK, and GLP-1 are satiety regulatory hormones [5,6]. These hormones are episodic hormonal signals occurring in unison with episodes of eating. They signal satiation and satiety either via the vagus nerve (which connects the gut to the brain) or via blood perfusing the hypothalamus.

It is accepted that in response to weight loss, counter-regulatory adaptations develop in the appetite regulatory system, including the gastrointestinal hormones, defending impositions that promote a negative energy balance. Different weight loss intervention approaches are likely to cause different counter-regulatory adaptations in terms of the content and the magnitude of the response. There is some evidence suggesting that diet-induced weight loss is associated with a compensatory increase in total ghrelin (GT) plasma levels and a blunted postprandial release of PYY and GLP-1 [7,8]. Exercise-induced weight loss may increase the drive to eat, as shown by increased levels of acylated ghrelin (AG) and subjective feelings of hunger in fasting, but it may also improve satiety as evidenced by an increase in the late postprandial release of GLP-1 after exercise training [9].

Few studies have investigated the combined effect of exercise and dieting on appetite in children and adolescents. Based on the limited data, it appears that ghrelin levels increase after a weight reduction program, with no change in PYY levels [10-13]. Similarly, we have observed an increase in ghrelin concentrations after weight loss in adolescents who participated in an exercise and diet intervention for four weeks in a summer camp program in a previous study (unpublished data).

Recently, the effect of high altitude on appetite regulation has attracted researchers’ interest. It is a widely observed phenomenon that a high altitude can induce loss of appetite [14-16]. In many studies, loss of appetite and the resulting decrease in energy intake have been attributed to acute mountain sickness (AMS), symptoms of which include headache and anorexia. However, loss of appetite cannot merely be a by-product of AMS because anorexia and weight loss still persist when symptoms of AMS have subsided [17]. Furthermore, studies conducted in normobaric hypoxia chambers, where other environmental stressors associated with high altitude are eliminated (extreme cold and physical exertion), have demonstrated that hypoxia *per se* can cause reduced appetite and energy intake, and loss of body weight [18]. It has been suggested that the effect of hypoxia on appetite is mediated by the changes in gastrointestinal hormones [19-22].

It is perceivable that to promote success in longterm weight loss, hypoxia can be implemented in a traditional diet and exercise weight loss program, because the negative effect of hypoxia on appetite might be able to balance the positive effect of diet and exercise. Exercise and hypoxia have been used in combination in sports to induce maximal increase in aerobic endurance in athletes, but have rarely been used in weight loss, especially in children and adolescents. We have run several sessions of weight loss summer camps designed for obese adolescents in Shanghai, China. The effect of exercise and hypoxia on weight loss has been explored in our preliminary studies and interesting results have been generated [23,24]. As such, we are now designing a randomly controlled trial to systematically investigate the longterm weight loss effect of exercise with hypoxia in obese adolescents and determine the mediating effect of the gastrointestinal hormones. We hypothesize that: 1) exercise and hypoxia will have an additive effect on weight loss via increasing energy expenditure and suppressing appetite; 2) hypoxia use will lead to less rebound of weight loss after intervention due to its potential negating effects on compensatory changes in appetite elicited by exercise and diet in a traditional weight loss program; and 3) changes in gastrointestinal hormones (including ghrelin, PYY, CCK, and GLP-1), as well as in cytokine interleukin (IL)-6, will be associated with changes in body weight after the intervention and during follow-up.

### Aims

The aims of this randomized controlled trial (RCT) are to evaluate the effectiveness of intermittent hypoxia and exercise, in combination with a balanced diet, on inducing short- and longterm fat loss in Chinese children and adolescents, and to determine the molecular mechanisms behind the benefits of hypoxia in enhancing weight loss.

## Methods/Design

### Design

**Figure 1:**
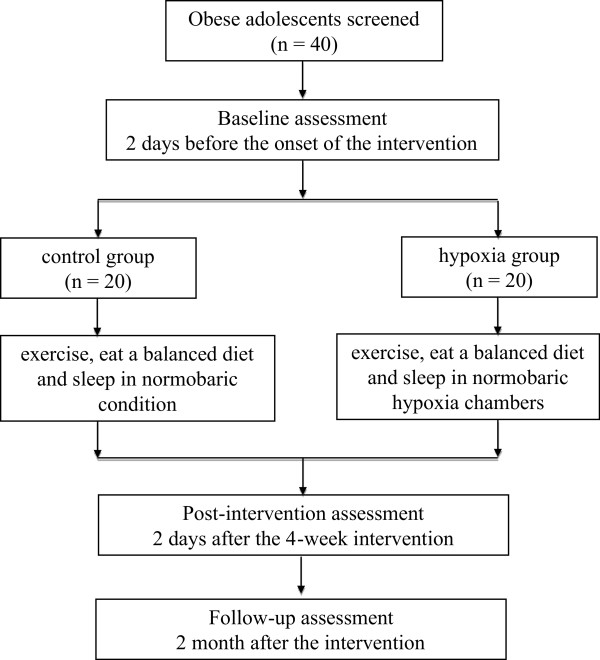
Flow of participants through the trial.

This study is a randomized controlled clinical trial with two parallel arms involving 40 obese adolescents. To assess the effectiveness of four weeks of intermittent hypoxia added to a traditional exercise and diet intervention on inducing short (after the four-week intervention) and longterm (at two months follow-up) weight loss in obese adolescents, we will recruit 20 adolescent boys and 20 adolescent girls (aged 11 to 15 years) from our summer weight loss camp. They will be stratified according to gender and randomly assigned to two groups: the control group who will exercise, eat a balanced diet, and sleep in a normobaric condition, and the hypoxia group, who will exercise, eat a balanced diet, and sleep in normobaric hypoxia chambers (‘sleep high and train low’). Outcome assessment and data analysis will be performed by trained professionals who will be blinded to the group assignment of subjects. The flow of participants through the trial is shown in Figure 1.

### Sample size estimation

Sample size estimation in this RCT is based on the expected fat loss following four weeks of hypoxia exposure plus exercise and dieting. The data of our preliminary experiment showed that the means of fat percentage decrease in the normoxia and hypoxia groups were 3.1 and 6.0% respectively, and the standard deviation of the change was about 3%. It is estimated that a sample size of 17 participants per group will be required to observe a similar result with a power of 80%. Considering a 15% dropout and exit rate, we will recruit 40 subjects with 20 in each group.

### Ethical approval and consent

The study will be conducted according to the principles expressed in the Declaration of Helsinki. The study protocol has been approved by the institutional review board at the Shanghai University of Sport (reference number: 2014 Ethics Approval Note 1). Participants in this study are volunteers. None of the measurements or the intervention are known to entail any significant health risk. The study has its own physician to ensure the eligibility and safety of all participants. All data will be handled and archived confidentially. The benefits and associated risks of the study will be carefully explained and the voluntary nature of participation will be emphasized. Informed consent and assent will be obtained from all participants and their parents or legal guardians. Participants and their parents or legal guardianswill have the option to end the participation at any stage if they so wish. If the physician and the principal investigator believe that there are risks of serious adverse events in the study the trial will be stopped. The trial is registered with Chinese Clinical Trial Registry as ChiCTR-TRC-14004106.

### Subject recruitment

Obese adolescents aged 11 to 15 years will be recruited from the children registered for the 2014 summer weight loss camp at the Shanghai University of Sport. A public health nurse will assess the Tanner stage of each subject using the Tanner grading system [25,26]. Obesity will be defined based on body-mass index (BMI), calculated as weight in kilograms divided by height in meters squared (kg/m^2^). Although a BMI of ≥ 25 kg/m^2^ and ≥ 30 kg/m^2^ are international cutoff points for overweight and obesity, for many Asian populations individuals with a BMI of ≥ 23 kg/m^2^ are considered to be at increased risk and those with a BMI of ≥ 27.5 kg/m^2^ at high risk [27]. Thus, we chose a BMI of ≥ 25 kg/m^2^ as the criterion to recruit obese adolescents. Adolescents will be excluded if they have concomitant renal, hepatic, or cardiac disease, and/or are being treated with drugs that could affect body weight and appetite (such as orlistat, lorcaserin, and phentermine-topiramate, as well as appetite suppressants).

### Randomization and blinding

After a participant is confirmed to be eligible and written informed consent has been obtained, she/he will be randomly assigned to the hypoxia or normoxia group. The randomization procedure, stratified according to gender, will be conducted by an independent statistician using a computerized randomization program. In order to minimize the potential bias, the exercise physiologist and the dietitian who manage the exercise training and the diet intervention will be blinded as to whether a subject will sleep in the hypoxia chamber or not. The hypoxia chamber will be prepared by an independent researcher. The researcher performing outcome assessments will be blinded to the subjects’ intervention allocation.

### Intervention

All the subjects, including both the normoxia (control) and the hypoxia groups, will undergo four weeks of aerobic exercise training and dieting (eating a balanced diet). In addition, the normoxia group will sleep in normal conditions while the hypoxia group will sleep in a normobaric hypoxic chamber every night. All the measurements will be conducted two days prior to, and post-intervention, and repeated two months later after the intervention (two months follow-up). Fasting blood samples will be collected in the morning of the testing day. In order to maximize the compliance and avoid the occurrence of any accident the intervention will be closely supervised by a physician, a dietitian, and an exercise physiologist.

### Exercise training

The same aerobic exercise training will be applied to both the normoxia and the hypoxia groups. Participants will exercise for six days per week, twice daily, for one hour per session. The intensity of the exercise will be estimated using the Metabolic Equivalent of Task (MET) score. MET, the unit of energy expenditure, will be obtained by dividing oxygen uptake values (ml · kg^-1^ · min^-1^) by 3.5 (1 MET is defined as resting metabolic rate that is 3.5 ml · kg - 1 · min - 1). VO_2_ will be measured using Cosmed K4b2 Portable Metabolic Measurement System (Cosmed, S.r.l., Rome, Italy) according to the manufacturer’s instructions.To promote participants’ interest, the exercise training will consist of three different activities including swimming (intensity: 6 MET), aerobic exercise (intensity: 7.5 MET), and basketball (intensity: 6 MET).

### Diet modification

All participants will receive well-defined and balanced daily meals during the four-week intervention. Dietary recommendations will be individualized based on the individual’s basal metabolic rate and will range from 1,600 to 2,000 kcal/day. The basal metabolic rate will be calculated using the Mifflin equation [28]. The caloric intake will be calculated based on the Chinese food chart. Each day, three well-balanced meals will be provided with the following calorie allocations: breakfast 35%, lunch 40%, and dinner 25%. Each meal comprises 30% protein, 20% fat, and 50% carbohydrate by energy. Animal and vegetable oil and starch-rich food will be minimized, while the intake of vegetables, fruits, bean products, rabbit meat, beef, pork, and cellulose will be increased. This prescribed diet includes pivotal nutrients such as vitamins, minerals, essential amino acids, fiber, and polyunsaturated fatty acids.

### Hypoxia exposure

To test the hypothesis that hypoxia exposure would ameliorate the compensatory increase in appetite elicited by exercise and diet, the experimental group (hypoxia group) will sleep in a normobaric hypoxia environmental chamber every night during the four- week intervention. The advantage of using an environmental chamber compared to real altitude situations is that the effects of hypoxia can be isolated from the influence of other confounding factors present in a real altitude situation, such as the influence of temperature, humidity, and physical activity levels. The normobaric hypoxia will be designed to mimic an altitude of 2700 m. We will use the large hypoxic training system (Low Oxygen System, Dubai, Germany)) of the hypoxia test laboratory of Shanghai Oriental Oasis Training Base to simulate a hypoxic environment (14.7% O2; approximately 2700 m). After a one-day hypoxia acclimation period, participants of the hypoxia group will sleep in the hypoxia training laboratory for 10 hours every night (from 21:00 to approximately 7:00 the next day), seven times per week, for four weeks.

### Attrition and compliance

Due to the voluntary nature of the enrollment of the summer weight loss camp, attrition rates will be very low. In a pilot trial involving 50 overweight male Chinese adolescents, 47 children completed a four-week diet and exercise intervention, and only three children (6%) dropped out of the study due to loss of interest. Considering the addition of a two-month follow-up, we expect a higher attrition rate (15%) in this study. Our recruitment plan was made based on this consideration.

### Outcome assessments

Baseline assessments will be conducted two days before the onset of the four-week intervention. Post-intervention and two month follow-up assessments will be conducted two days and two months after completion of the intervention, respectively. The primary outcome of the study will be body composition and the secondary outcome measures will include appetite score and blood levels of gastrointestinal hormones including leptin, ghrelin, PYY, CCK and GLP-1, as well as circulating levels of IL-6. All the assessments will be performed in the laboratory of exercise physiology at the Shanghai University of Sport.

### Primary outcome

#### Body composition

Body mass and height will be measured using a digital scale (Yaohua Weighing System Co., Shanghai, China) and a wall-mounted stadiometer (TANITA, Tokyo, Japan), respectively. Body composition and fat distribution will be measured using dual energy X-ray absorptiometry (DXA) (GE Lunar Prodigy, Madison, Wisconsin, United States)). The software (ENCORE, version 10.50.086, GE, Madison, Wisconsin, United States)) will be used to analyze total lean mass (TLM), total fat mass (TFM), total body fat percentage (%TBF), android fat percentage (%AF), and gynoid fat percentage (%GF).

### Secondary outcomes

#### Cardiorespiratory fitness

Peak oxygen consumption (VO_2peak_) for all participants will be measured using the Cosmed (K4b2) portable metabolic system. Expired respiratory gases will be collected on a breath-by-breath basis during a submaximal treadmill (H/P/Cosmos Pulsar 4.0, Cosmos Sports & Medical Ltd., Nussdorf- Traunstein, Germany) test. Exercise will start at a speed of 2 km/h, which will be increased every 2.5 minutes by 1 km/h until 8 km/h is reached. The criterion of exercise termination will be 80% of the maximum heart rate (HR_max_). Trained research assistants will record heart rate and power output data at the end of each stage. Heart rate will be measured using a polar heart rate monitor (Polar Electro, Kempele, Finland).

#### Blood analyses

Fasting blood samples (2 mL, 12-hour fasting) will be obtained at baseline, post-intervention, and at two months follow-up. Serum levels of gastrointestinal hormones including leptin, ghrelin, PYY, CCK and GLP-1, as well as cytokine IL-6, will be measured using commercially available ELISA kits (R&D Systems, Minneapolis, Minnesota, United States). As reported, the lower limit of sensitivity of the assays will range between 0.8 pg/mL and 10 pg/ml. The intra-assay coefficient of variation (CV) will be less than 5% and the inter-assay CV will be less than 10%. Absorbance will be read at 450 nm wavelength using a microplate reader (Bio-Rad 550, Bio-Rad, Hercules, California, United States).

#### Appetite assessment

Children’s appetite at baseline, during, and post-intervention, and during follow-up will be assessed via a simple eight-item appetite questionnaire (Additional file 1). The questionnaire was developed by modifying the eight-item Council on Nutrition Appetite Questionnaire (CNAQ). The CNAQ is a short, simple, appetite assessment tool, which has been validated and proved to be able to predict weight loss in community-dwelling adults and nursing home residents [29]. We modified the CNAQ to reflect the nature of our intervention.

### Data management and statistical analysis

Data generated in the study will be collected and summarized using mean (standard deviation) values. Group differences in demographical and clinical characteristics at baseline will be tested using a two-sample t-test for quantitative data and chi-square test for qualitative data. Repeated measures analysis of variance (RM ANOVA) will be used to analyze the main effect of treatment and potential time-by-treatment interactions. For these analyses, parametric assumptions of the normality and homoscedasticity will be checked using standard tests and graphical methods. If those assumptions are violated, data transformation and non-parametric procedures will be considered sequentially. Multivariate regression analysis will be performed to analyze the relationship between changes in body composition, appetite score, and gastrointestinal hormones following intervention and during follow-up. Multiple models will be developed and tested for different sets of outcome measures and regressor variables. All statistical analyses will be performed using the Statistical Package for the Social Sciences (SPSS, Inc., Chicago, Illinois, United States) for Windows version 18.0, and a significance level of 0.05 will be used.

## Discussion

To the best of our knowledge, our study will be the first to evaluate the effectiveness of ‘sleep high and train low’ on short- and longterm weight loss among obese adolescents. The study will focus on obese adolescents due to the rising prevalence of obese children in China and the adverse impact of childhood obesity on adult health. Adolescent obesity has been attributed mainly to a sedentary lifestyle and an unhealthy diet. In China, a great effort has been made to use exercise and dietary intervention to treat obesity and metabolic disorders in children. However, the weight rebound following most of the weight loss programs has made such an effort fruitless. The weight rebound is believed to be caused by the compensatory increase in appetite induced by exercise and diet. Conceivably, the agent that can counteract this compensatory effect will benefit obese patients in achieving longterm weight loss. Hypoxia is potentially one of such agents: the anorexia effect of hypoxia has been widely observed and might be utilized to dampen the appetite compensatory effect of exercise and diet, thereby promoting the success of weight loss in the longterm. Due to the profound benefits of exercise training and diet in the obese population, we do not expect to use hypoxia as a replacement, but rather as a supplement to exercise and diet intervention. Therefore, children in the control group will undergo the same exercise training and diet as the hypoxic group in order to evaluate the value of hypoxia in promoting weight loss, and minimizing weight rebound after intervention.

Instead of using continuous hypoxic exposure such as high altitude, we choose to use intermittent hypoxic exposure, which has been recommended as an option for the free-living obese population [30]. The effect of intermittent hypoxia exposure on weight reduction has been reported previously. In a model of diet-induced obese mice, it has been shown that intermittent hypoxic exposure induced decreases in body mass, blood glucose levels, and cholesterol erythropoietin concentrations [27,31]. In a few human studies, the synergistic effect of exercise and hypoxia in treating obesity and associated metabolic disorders has been demonstrated, where low intensity exercise training at normobaric hypoxic condition (15% O_2_) led to more weight loss and greater improvement in metabolic health compared to training at ambient conditions (21% O_2_) [32-34]. However, further evidence-based studies are needed to carefully evaluate the therapeutic value of hypoxia and the model of utilization in treating obesity, especially in children. Intermittent hypoxia exposure, as used in the ‘sleep high and train low’ paradigm has been proposed for endurance athletes to maximally enhance their endurance exercise capabilities, however the clinical use of intermittent hypoxia in the management of cardiometabolic diseases has rarely been tested. Our study will be the first to evaluate the effect of ‘sleep high and train low’ on weight loss in obese adolescents. Through monitoring a wide spectrum of health indices including blood pressure, heart rate, vital capacity, blood glucose and insulin levels, blood lipid profile, and immune function we will careful monitor any possible side effects associated with hypoxia exposure.

Furthermore, we will investigate the mechanism for the effect of hypoxia on weight management. We will focus on the regulatory effect of ‘sleep high and train low’ on gastrointestinal hormones levels. In addition, we hypothesize that the cytokine IL-6 might also play a role in mediating the appetite regulatory effect of exercise (positive effect) and hypoxia (negative effect). The clarification of mechanisms leading to weight loss in ‘sleep high and train low’ might provide information for the development of new strategies in combating obesity in the future.

## Trial status

A pilot study involving a small group of overweight children has been completed based on the summer weight loss camp at our institution in 2013. The intervention modality has proven to be tolerated very well among children and has been effective in inducing significant weight loss. For the official trial, participant recruitment will start in April 2014. Baseline measurements will be taken in June 2014, and the four-week intervention will be completed by August 2014, which will be followed by a two month follow-up. Feedback on the preliminary results of participants’ health status will be provided to the participants upon completion of the study.

## Abbreviations

AG: Acylated ghrelin; AMS: Acute mountain sickness; CCK: Cholecystokinin; CNAQ: Council on Nutrition Appetite Questionnaire; CV: coefficient of variation; GLP-1: Glucagon-like peptide-1; IL6: Interleukin-6; MET: Metabolic Equivalent of Task; BMI: body-mass index; PYY: Peptide YY; RCT: randomized controlled trial; TG: Total ghrelin.

## Competing interests

The authors declare that they have no competing interests.

## Authors’ contributions

RW and PC conceived the study, participated in its design and coordination, and drafted the manuscript. WX, NW and BG carried out the pilot study and helped in the design of the current study. DL and XW participated in the design of the study and helped to draft the manuscript. All authors read and approved the final manuscript.

## Supplementary Material

Additional file 1 Appetite questionnaire. Click here for file
