# Supplementary material for: The effect of ‘sleep high and train low’ on weight loss in overweight Chinese adolescents: study protocol for a randomized controlled trial
Source: Trials. 2014 Jun 25;15:250. doi: 10.1186/1745-6215-15-250 (PMC4082418; doi:10.1186/1745-6215-15-250)
Supplement: Additional file 1 — Appetite questionnaire. [file 1745-6215-15-250-S1.docx]

**Additional File 1.**

Appetite questionnaire

Name: _____________________________________________ Sex (circle): Male Female

Age: ________________ Weight: _____________________ Height: _________________

Date: __________________________

Administration Instructions: Ask the subject to complete the questionnaire by circling the correct answers and then tally the results based upon the following numerical scale: a 1, b 2, c 3, d 4, e 5. The sum of the scores for the individual items constitutes the appetite score.

**1. My appetite is**

a. very poor

b. poor

c. average

d. good

e. very good

**2. When I eat**

a. I feel full after eating only a few mouthfuls

b. I feel full after eating about a third of a meal

c. I feel full after eating over half a meal

d. I feel full after eating most of the meal

e. I hardly ever feel full

**3. I feel hungry**

a. rarely

b. occasionally

c. some of the time

d. most of the time

e. all of the time

**4. Food tastes**

a. very bad

b. bad

c. average

d. good

e. very good

**5. Compared to before weight loss program, food tastes**

a. much worse

b. worse

c. just as good

d. better

e. much better

**6. Normally I eat**

a. less than one meal a day

b. one meal a day

c. two meals a day

d. three meals a day

e. more than three meals a day

**7. I feel sick or nauseated when I eat**

a. most times

b. often

c. sometimes

d. rarely

e. never

**8. Most of the time my mood is**

a. very sad

b. sad

c. neither sad nor happy

d. happy

e. very happy
